# Systemic glucose variability predicts cerebral metabolic distress and mortality after subarachnoid hemorrhage: a retrospective observational study

**DOI:** 10.1186/cc13857

**Published:** 2014-05-04

**Authors:** Pedro Kurtz, Jan Claassen, Raimund Helbok, J Michael Schmidt, Luis Fernandez, Mary Presciutti, R Morgan Stuart, E Sander Connolly, Kiwon Lee, Neeraj Badjatia, Stephan A Mayer

**Affiliations:** 1 Neurological Intensive Care Unit, Milstein Hospital 8 Center, 177 Fort Washington Ave., New York, NY 10032, USA

## Abstract

**Introduction:**

Cerebral glucose metabolism and energy production are affected by serum glucose levels. Systemic glucose variability has been shown to be associated with poor outcome in critically ill patients. The objective of this study was to assess whether glucose variability is associated with cerebral metabolic distress and outcome after subarachnoid hemorrhage.

**Methods:**

A total of 28 consecutive comatose patients with subarachnoid hemorrhage, who underwent cerebral microdialysis and intracranial pressure monitoring, were studied. Metabolic distress was defined as lactate/pyruvate ratio (LPR) >40. The relationship between daily glucose variability, the development of cerebral metabolic distress and hospital outcome was analyzed using a multivariable general linear model with a logistic link function for dichotomized outcomes.

**Results:**

Daily serum glucose variability was expressed as the standard deviation (SD) of all serum glucose measurements. General linear models were used to relate this predictor variable to cerebral metabolic distress and mortality at hospital discharge. A total of 3,139 neuromonitoring hours and 181 days were analyzed. After adjustment for Glasgow Coma Scale (GCS) scores and brain glucose, SD was independently associated with higher risk of cerebral metabolic distress (adjusted odds ratio = 1.5 (1.1 to 2.1), *P* = 0.02). Increased variability was also independently associated with in hospital mortality after adjusting for age, Hunt Hess, daily GCS and symptomatic vasospasm (*P* = 0.03).

**Conclusions:**

Increased systemic glucose variability is associated with cerebral metabolic distress and increased hospital mortality. Therapeutic approaches that reduce glucose variability may impact on brain metabolism and outcome after subarachnoid hemorrhage.

## Introduction

Hyperglycemia has been associated with morbidity and poor outcome in patients with subarachnoid hemorrhage [1-4]. Tight glucose control with intravenous insulin has been shown to reduce mortality among surgical ICU patients [5,6], but not in mixed populations of critically ill patients [7-10]. The impact of tight glycemic control in neurological critically ill patients remains controversial. While some data suggest that intensive insulin therapy fails to improve the outcome of neurologic patients and may be deleterious due to an increased incidence of hypoglycemia and low brain tissue glucose levels, some authors have shown that tighter glycemic control may avoid neurological complications in the ICU [1-5,11-13]. Microdialysis studies of cerebral metabolism indicate that tight glucose control is associated with an increased risk of metabolic distress, which is defined as an elevation of the lactate/pyruvate ratio [1,14-22].

Cerebral energy production depends on an adequate supply of glucose. Systemic glucose levels affect glucose availability to the brain and can impact cellular metabolism and energy production after subarachnoid hemorrhage (SAH). Because of impaired glucose transport, systemic glucose levels considered to be normal may be relatively insufficient to meet the increased cerebral metabolic demand seen in patients with SAH [1,16,20].

Both hypoglycemia and hyperglycemia have been shown to exacerbate secondary brain injury [2-4,16,20,21,23] after SAH. Acute fluctuations of systemic glucose have also been associated with oxidative stress in diabetic outpatients [24,25], with increased mortality in critically ill patients and with worse functional outcome and mortality in neurological patients [26-32]. Patients with SAH may be more vulnerable to glycemic variability if these acute fluctuations trigger cerebral metabolic distress and lead to secondary brain injury.

In this study, we sought to understand better the potential role of increased systemic glucose variability in cerebral oxidative metabolism and potentially secondary brain injury. Specifically, we hypothesized that increased glycemic variability is associated with cerebral metabolic distress and increased mortality in patients with SAH.

## Materials and methods

### Patients

We retrospectively reviewed 28 consecutive patients admitted to the neurological ICU at Columbia University Medical Center between May 2006 and January 2009 after SAH who underwent multimodality neuromonitoring with intracranial pressure (ICP), cerebral microdialysis and brain tissue oxygen pressure (PbtO2) as part of their clinical care. This study was approved by the Columbia University Institutional Review Board (IRB). Written informed consent was obtained from all patients or person responsible.

### Clinical management

Patient care for SAH conformed to guidelines established by the American Heart Association [11]. Hemodynamic and fluid management were targeted to maintain cerebral perfusion pressure (CPP) >60 mm Hg and ICP <20 mm Hg. Hemoglobin cutoff for blood transfusions was 8 g/dL unless there was clinical, imaging or laboratory evidence of active cerebral or myocardial ischemia. Fever was aggressively treated using intravascular (Celsius Control System®, Innercool Therapies, Inc, San Diego, CA, USA) or surface (Arctic Sun Cooling System®, Medivance Inc, Louisville, CO, USA) cooling devices. Shivering was treated with buspirone, skin counterwarming, magnesium infusion and analog-sedation (dexmedetomidine, fentanyl or meperidine) according to a stepwise protocol [33,34].

### Systemic glucose control

Systemic glucose was measured with the Sure Step Flexx system (Lifescan, Milpitas, CA, USA) using arterial blood and the target range was between 4.4 and 8.3 mmol/L (80 to 150 mg/dL) as part of a glucose control protocol using intravenous insulin infusion Humulin (©Lilly, Indianapolis, IN, USA). Hypoglycemia with systemic glucose below 3.3 mmol/L (60 mg/dL) was managed with a bolus of 20 to 25 g of glucose in D50 solution. Enteral nutrition (Osmolite, Ross Nutrition, Abbott Laboratories, Columbus, OH, USA) was provided via a naso-duodenal tube starting within the first 24 hours of admission, aiming to 25 kcal/kg/day of ideal body weight. No parenteral nutrition was given. Almost all of the systemic glucose measurements while patients underwent neuromonitoring were performed hourly. The median number of systemic glucose measurements per patient was 105 (interquartile range (IQR), 69 to 144).

### Multimodality neuromonitoring

ICP, PbtO2 and microdialysis probes were placed via a triple lumen bolt at the bedside using full sterile technique. ICP was measured using an intraparenchymal fiberoptic catheter (Camino System, Integra Neurosciences®, Plainsboro, NJ, USA). Hourly microdialysis samples were obtained with a 10 mm membrane length CMA-70 microdialysis catheter (CMA Microdialysis®, Stockholm, Sweden). The probes were placed via a frontal approach into the hemisphere deemed at greatest risk for secondary injury (that is, perihematomal or pericontusional tissue, or the ipsilateral anterior watershed zone in lateralized SAH) or in the right frontal lobe in patients with diffuse injury. Immediately after the procedure, a brain CT scan was performed in each patient to confirm the location of the microdialysis catheter.

### Cerebral microdialysis

A CMA 106 microdialysis perfusion pump (CMA Microdialysis®) was used to perfuse the interior of the catheter with sterile artificial cerebrospinal fluid (Na^+^ 148 mmol/L, Ca^2+^ 1.2 mmol/L, Mg^2+^ 0.9 mmol/L, K^+^ 2.7 mmol/L, Cl^-^ 155 mmol/L) at a rate of 0.3 μl/minute. Samples were collected every 60 minutes into microvials, and immediately analyzed at the bedside for glucose, lactate and pyruvate (mmol/L) with the CMA 600 analyzer (CMA Microdialysis®). At least one hour passed between the insertion of the probe and the start of the sampling, to allow for normalization of changes due to probe insertion. The analyzer was automatically calibrated on initiation and every six hours using standard calibration solutions from the manufacturer. Quality controls at three different concentrations for each marker were performed daily.

### Physiologic variables

Physiological variables including heart rate (HR), arterial blood pressure, respiratory rate (RR), fraction of inspired oxygen (FiO2) and oxygen saturation (SpO2) were continuously monitored in all patients. Hourly ICP and mean arterial pressure (MAP) were prospectively recorded as part of the standard of care. CPP was calculated as CPP = MAP – ICP, with both MAP and ICP referenced to the level of the foramen of Monroe. FiO2 was routinely maintained at 40%. Symptomatic vasospasm was defined as neurologic worsening and/or cerebral infarction attributed to vasospasm.

### Glycemic variability and metabolic distress

Daily glycemic variability was assessed using standard deviation (SD) [29,35-37]. SD is calculated as the squared root of the average of the squared differences between individual glucose values and the mean. SD was calculated daily to test for associations with metabolic distress and calculated for the entire monitoring period to test for associations with mortality.

Metabolic distress was defined as a lactate/pyruvate ratio (LPR) above 40. This threshold was defined based on previous reports demonstrating associations with cerebral metabolic disarray, cerebral ischemia, or poor clinical outcome in patients with SAH [38,39].

### Data acquisition

A Solar 8000i utilizing a General Electric Medical Systems Information Technologies’ Unity Network® was used as the patient physiologic monitor. A high resolution data acquisition system (BedmasterEX, Excel Medical Electronics, Jupiter, FL, USA) using an open architecture of the Unity Network® automatically acquired vital signs, alarm and waveform data from all the patient monitoring devices in the NICU. Digital data were acquired every five seconds and recorded in an SQL database. Waveform data were stored at a resolution of 240 Hz in binary files. LICOX® (Integra Neuroscience, Plainsboro, NJ, USA) and brain metabolism data were incorporated into the data acquisition system utilizing the communications (COM) port on the device which was plugged into a serial-to-TCP/IP interface device (Equinox ESP-8, Avocent, Sunrise, FL, USA).

### Statistical analysis

Due to the small sample of patients and large number of measurements the data were analyzed using generalized estimating equations (GEE). Univariate analyses were used to test for associations between predictor and outcome variables. Variables with significant associations (*P* <0.1) were considered candidates for the multivariable analyses. Multivariable models were constructed using a general linear model (GLM) with a logistic link function (logistic regression), extended by generalized estimating equations (GEE) to account for within-subject variation. The within-subject correlation structure was modeled using the auto-regressor of the first order (AR-1) [40-42]. Model building was performed with a stepwise procedure starting with the variable of interest. The relationship between serum glucose variability (SD) and cerebral metabolic distress was assessed using a multivariable model. The occurrence of at least one episode of metabolic distress (LPR >40) in each day of monitoring was considered a binary outcome variable. SD was tested as the main predictor variable and adjusted for significant covariates. We reported the final multivariable model. The model building procedure used the corrected quasi-likelihood under independence model criterion (QICC) for model selection [40].

Finally, in order to identify independent associations between SD and outcome we fitted a multivariable logistic regression model with hospital mortality as the binary outcome. Serum glucose variability averaged over the period of monitoring was entered as the predictor variable and adjusted for other significant covariates and clinically important variables. Goodness of fit was assessed with the Hosmer-Lemeshow test.

Adjusted odds ratios (OR) and 95% confidence intervals (CI) were reported for all significant predictor variables. All statistical analyses were performed using SPSS 16 software (SPSS Inc., Chicago, IL, USA). A *P* value <0.05 was considered statistically significant.

## Results

### Clinical characteristics and systemic parameters

Patients’ baseline characteristics are listed in Table 1. All 28 patients included in the study were mechanically ventilated and had a GCS less than or equal to 8 at the time of monitoring. During the study period, 3,139 hourly microdialysate samples and serum glucose measurements were collected (median per patient 105 hourly samples (IQR, 69 to 144). Serum glucose variability was calculated for each of the 181 days of neuromonitoring. The median duration from admission to the start of neuromonitoring was two days and the median duration of monitoring was six days. Values for multimodality monitoring including CPP, PbtO_2_, systemic glucose and hemoglobin concentrations, as well as SD are presented in Table 2. We did not record other systemic parameters that may influence brain metabolism such as pCO2 and temperature.

**Table 1:** Clinical characteristics (number = 28)

**Variable**	**Median or number**	**IQR or%**
Age	54	41 to 61
Gender (female)	19	68
Diabetes mellitus	3	11
Hunt Hess		
2	1	4
3	5	18
4	8	29
5	14	50
Modified Fisher		
2	4	14
3	14	50
4	10	36
APACHE II	23	19 to 29
Admission Glasgow Coma Scale (GCS)	6	5 to 9
Days from admission to neuromonitoring	2	1 to 4
Days with neuromonitoring	6	4 to9
Delayed cerebral ischemia (DCI)	10	36
Symptomatic vasospasm	7	25
Hospital mortality	7	25

Data are reported as number (%) or median (interquartile range) unless otherwise indicated. APACHE II, Acute Physiology and Chronic Health Evaluation II; IQR, interquartile range.

**Table 2:** Multimodality monitoring

**Variable**	**Median**	**IQR**
Cerebral perfusion pressure (mmHg)	95	78 to 105
Hemoglobin (g/dL)	9.7	9 to 10.5
Serum glucose (mmol/L)	7.7	6.9 to 8.3
Serum glucose variability		
Standard deviation (SD) per day	1.4	1.2 to 1.8
Microdialysis		
Lactate (mmol/L)	4.0	3.1 to 4.8
Pyruvate (mmol/L)	121	87 to 162
Glucose (mmol/L)	0.98	0.68 to 1.48
LPR	30	27 to 50
PbtO2 (mmHg)	28	20 to 40

IQR, interquartile range; LPR, lactate/pyruvate ratio; PbtO2, partial pressure of brain tissue oxygen.

### Hypoglycemia

No episodes of severe hypoglycemia (<2.3 mmol/L) occurred during the study period. Sixteen patients (57%) presented with at least one episode of moderate hypoglycemia (<3.9 mmol/L). Ten of these patients had one or two episodes and the maximum number of episodes occurred in one patient – five episodes. There was no difference in the number of episodes of moderate hypoglycemia sustained by patients with increased SD (above the median) as compared to those with lower SD (1 IQR (0 to 3) versus 0 IQR (0 to 1.5)); *P* = NS, respectively). The development of moderate hypoglycemia was also tested in the multivariate models for metabolic distress and hospital mortality but no association was found.

### Glycemic variability and metabolic distress

**Figure 1:**
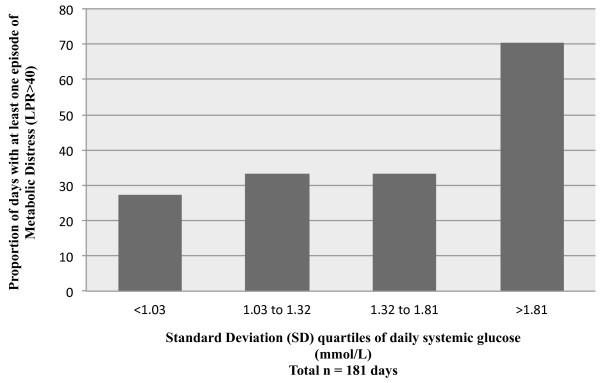
**Relative frequency of at least one episode of metabolic distress (LPR >40) per day monitored across the quartiles of daily standard deviation (SD).** The multivariable general linear model (GLM) with a logistic link function using GEE showed an independent association between SD and metabolic distress. GEE, generalized estimating equations.

SD was treated as a continuous variable in the multivariable model with a binary outcome variable: at least one episode of metabolic distress per day. The proportion of days with at least one episode of metabolic distress progressively increased with SD (Figure 1). After adjusting for GCS and brain glucose, SD was independently associated with an increased risk of developing at least one episode of metabolic distress per day (Table 3).

**Table 3:** Predictors of at least one episode per day of metabolic distress (LPR >40)

		**Univariate analysis**		**Multivariate analysis**		
**Variable**	**Threshold**	**Unadjusted OR**	**CI**	**Adjusted OR**	**CI**	***P *** **value**
Glucose variability (SD)	NA	1.3	0.9 to 1.6	1.5	1.1 to 2.1	0.02
Brain glucose	NA	0.4	0.2 to 0.8	0.3	0.1 to 0.8	0.02
Glasgow Coma Scale	NA	0.8	0.7 to 0.9	0.7	0.6 to 0.9	<.001

Multivariable logistic regression model accounting for between-subject and within-subject variations over time using generalized estimating equations (GEE) adjusted for the variables listed. All variables, including SD were entered as continuous variables. CI, confidence interval; NA, not applicable; OR, odds ratio; SD, standard deviation.

### Glycemic variability and outcome

**Figure 2:**
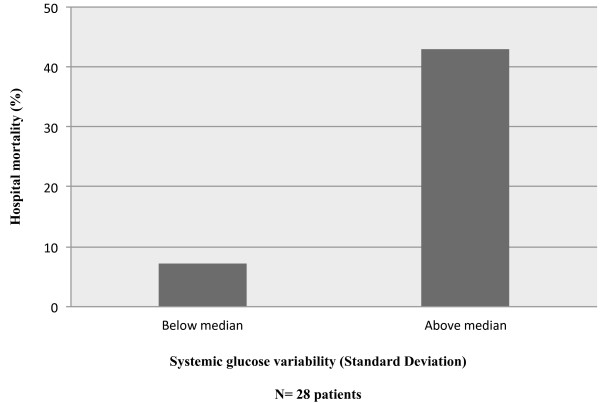
**Hospital mortality of patients with serum glucose variability below and above the median (median = 1.4) for standard deviation (SD).** Multivariable logistic regression demonstrated independent associations between SD and hospital mortality.

Hospital mortality was higher for patients with increased variability (SD above the median) (Figure 2). After adjusting for age, worst Hunt Hess on admission, daily GCS and the development of delayed cerebral ischemia (DCI), SD was independently associated with increased hospital mortality in a multivariable logistic regression model (Table 4).

**Table 4:** Predictors of hospital mortality

		**Univariate analysis**		**Multivariate analysis**		
**Variable**	**Threshold**	**Unadjusted OR**	**CI**	**Adjusted OR**	**CI**	***P *** **value**
Glucose variability (SD)	NA	5.9	0.9 to 37	10.4	1.3 to 86	.03
GCS	Every 1 point	1.1	0.8 to 1.4	0.5	0.2 to 0.99	.04

Multivariable logistic regression model with hospital mortality as the binary outcome adjusted for the variables listed, age, Hunt Hess, and DCI. GCS and SD were entered as continuous variables. CI, confidence interval; DCI, delayed cerebral ischemic; GCS, Glasgow Coma Scale; NA, not applicable; OR, odds ratio; SD, standard deviation.

We tested for interactions between DCI, diabetes status and systemic glucose and SD and no interaction was found. Furthermore, although our cohort had only three diabetic patients we compared systemic glucose and SD between patients with and without DM and no difference was found (median systemic glucose 7.7 IQR (7.2 to 8.7) versus 7.6 IQR (6.9 to 8.2); *P* = NS and median SD 1.9 (IQR 1.3 to 1.9) versus 1.6 (IQR 1.4 to 2.0); *P* = NS, respectively).

## Discussion

In this study we demonstrated an association between increased systemic glucose variability with cerebral metabolic distress and mortality after SAH.

In our study we used metabolic distress to evaluate energy failure. Metabolic distress, defined as an elevated LPR above 40, has been reported in the absence of ischemia, possibly caused by mitochondrial dysfunction, seizures or reduced substrate availability. Moreover, elevated LPR is a well-studied complication and has been shown to be associated with poor outcome [16,34,38,39,43-48].

Systemic glucose variability has been associated with mortality in mixed populations of critically ill patients [29-31] and after traumatic brain injury [26,49]. Although variability has been shown to affect diabetic and non-diabetic patients differently [50], we found no effect in our cohort. Recently glucose variability has been associated with the development of cerebral infarction in a cohort of SAH patients [49]. In our study, daily acute fluctuation of systemic glucose was a predictor of the development of cerebral metabolic distress after adjusting for the presence of DCI. This finding suggests that increased glycemic variability and oxidative metabolism may be associated with, and contribute to, poor outcome. Interestingly, the occurrence of hypoglycemia was not associated with increased SD or mortality in our model. This may be explained by the absence of severe hypoglycemia during the study and the very low number of episodes of moderate hypoglycemia.

The potential mechanisms involved in our findings range from the well described morbidity of hypoglycemia and hyperglycemia to oxidative stress triggered by acute fluctuations of glucose levels [3,4,24]. In a case-control study of diabetic outpatients serum glucose variability showed a strong correlation with 8-iso prostaglandin F2, a marker of oxidative stress [24]. The pathophysiology behind this relationship is not clearly defined but potentially involves mitochondrial dysfunction caused by overproduction of superoxide by the mitochondrial electron-transport chain [51-53].

Our study has a number of important limitations. First, we were not able to analyze the temporal relation between the development of metabolic distress and glycemic variability, which limits any inference of causality. Second, we did not evaluate factors that might be related to variability and may influence its effect on outcome, such as intensive insulin therapy, sepsis and organ dysfunction. Third, brain glucose, lactate and pyruvate are involved in multiple biochemical pathways, being produced and consumed. This limits straightforward interpretation of their concentrations, especially as microdialysis only measures the extracellular pool. Fourth, we cannot assess mitochondrial dysfunction directly, which can cause abnormal oxidative metabolism in the presence of adequate oxygen and substrate delivery. Fifth, a glucometer was the method used in the study, which may add inaccuracy to systemic glucose measurement and potentially affect variability. Sixth, we were not able to provide some physiological parameters that may affect brain metabolism, such as body temperature and pCO2 levels. Finally, although we found an association with hospital mortality, we did not prospectively evaluate functional short and long-term outcomes, which will be critical for future studies in patients with SAH.

## Conclusions

We showed that glycemic variability is associated with cerebral metabolic distress and hospital mortality in SAH patients. Our findings are hypothesis generating but may have important clinical implications. With increasing evidence that systemic glucose variability is deleterious to critically ill neurological patients, strategies aimed at minimizing acute fluctuations may play a role in glycemic control protocols in the Neurological ICU. Further studies are needed in order to determine the effect of taking into account glycemic variability in glucose control protocols. Moreover, as multimodality monitoring becomes increasingly integrated into clinical practice, randomized clinical trials are needed to assess the effect of goal-directed interventions aimed at improving cerebral metabolic profiles on long-term outcomes of patients with SAH.

## Key messages

•Increased systemic glucose variability was independently associated with cerebral metabolic distress, as measured by microdialysis, in patients after poor-grade SAH.

•Glycemic variability was an independent predictor of mortality in patients with severe SAH.

•These findings suggest that glucose variability may impact cerebral oxidative metabolism and contribute to secondary brain injury.

## Abbreviations

AR-1: auto-regressor of the first order; CPP: cerebral perfusion pressure; DCI: delayed cerebral ischemia; GCS: Glasgow Coma Score; GEE: generalized estimating equations; GLM: general linear model; HR: heart rate; ICP: intracranial pressure; LPR: lactate pyruvate ratio; MAP: mean arterial pressure; PbtO2: partial pressure of brain tissue oxygen; SAH: subarachnoid hemorrhage; SD: standard deviation.

## Competing interests

The authors declare that they have no competing interests.

## Authors’ contributions

PK, JC, RH, NB, KL, ESC and SAM conceived of the study, participated in its design and coordination and helped to draft the manuscript. PK wrote the manuscript. PK, JC, RH, JMS, LF, MP and RMS collected the data and performed the statistical analysis. All authors critically reviewed, drafted and approved the manuscript for publication. All authors read and approved the final manuscript.
